# Retrieval Practice Fails to Insulate Episodic Memories against Interference after Stroke

**DOI:** 10.3389/fpsyg.2017.01074

**Published:** 2017-06-28

**Authors:** Bernhard Pastötter, Hanna Eberle, Ingo Aue, Karl-Heinz T. Bäuml

**Affiliations:** ^1^Department of Experimental Psychology, Regensburg UniversityRegensburg, Germany; ^2^Department of Neuropsychology, Bezirksklinikum RegensburgRegensburg, Germany

**Keywords:** stroke, memory impairment, retrieval practice, testing, interference

## Abstract

Recent work in cognitive psychology showed that retrieval practice of previously studied information can insulate this information against retroactive interference from subsequently studied other information in healthy individuals. The present study examined whether this beneficial effect of interference reduction is also present in patients with stroke. Twenty-two patients with stroke, 4.6 months post injury on average, and 22 healthy controls participated in the experiment. In each of two experimental sessions, participants first studied a list of items (list 1) and then underwent a practice phase in which the list 1 items were either restudied or retrieval practiced. Participants then either studied a second list of items (list 2) or fulfilled an unrelated distractor task. Recall of the two lists’ items was assessed in a final criterion test. Results showed that, in healthy controls, additional study of list 2 items impaired final recall of list 1 items in the restudy condition but not in the retrieval practice condition. In contrast, in patients with stroke, list 2 learning impaired final list 1 recall in both conditions. The results indicate that retrieval practice insulated the tested information against retroactive interference in healthy controls, but failed to do so in patients with stroke. Possible implications of the findings for the understanding of long-term memory impairment after stroke are discussed.

## Introduction

Stroke is often accompanied by residual cognitive impairments (Hochstenbach et al., 1998), commonly involving impairments of memory and attention, slowed information processing, and executive dysfunction (Lim and Alexander, 2009; Cumming et al., 2013). With regard to episodic memory, in general, stroke patients show reduced memory performance compared with healthy controls, with the prevalence of post-stroke memory impairment typically declining with increasing time after the stroke, ranging from about a 50% decline weeks after stroke to about a 10% decline one year after stroke (Snaphaan and De Leeuw, 2007). Stroke-related memory impairment can have a negative impact on a patient’s functional independence and social well-being, and his or her family’s daily life (Sturm et al., 2004), so that it is important to identify factors that may reduce stroke-related memory deficits and enhance memory and learning. One such factor may be retrieval practice, which has been shown to potentially enhance memory and learning in both healthy and clinical populations.

Retrieval practice can have a number of beneficial effects on memory and learning (Roediger et al., 2011). For instance, a very prominent benefit of retrieval practice, referred to as (backward) testing effect in the literature, is the finding that retrieval practice of previously studied information can improve its long-term retention more than restudy of the information does (e.g., Roediger and Karpicke, 2006; Karpicke and Roediger, 2008). The effect has been shown to be a robust phenomenon in both healthy and clinical populations, including persons with Alzheimer’s disease, multiple sclerosis (MS), and traumatic brain injury (TBI; e.g., Sumowski et al., 2010a,b; Small, 2012). Two (mutually non-exclusive) recent explanations of the (backward) testing effect are the semantic elaboration account and the episodic context account of retrieval-based learning. The semantic elaboration account assumes that testing of previously studied information improves its long-term retention because retrieval practice, more than restudy, induces elaborative or deep processing of the information (Carpenter, 2009). In contrast, the episodic context account of retrieval-based learning assumes that testing improves long-term retention because retrieval practice, more than restudy, enhances contextual processing of the practiced information (Karpicke et al., 2014).

Another prominent benefit of retrieval practice, referred to as the forward effect of testing in the literature (Pastötter and Bäuml, 2014), is the finding that retrieval practice of previously studied information can also increase retention of subsequently studied other information (e.g., Szpunar et al., 2008; Pastötter et al., 2011). The forward effect is striking because it is on the learning of new information that is not necessarily related to the retrieved information. The effect is robust and has been shown in both healthy individuals and persons with severe TBI (Pastötter et al., 2013). It has been attributed to both encoding and retrieval factors: retrieval practice compared to restudy has been suggested to enhance attention during the encoding of subsequently studied new information (Pastötter et al., 2011) and enhance contextual segregation between the previously studied and the new information at final test (Szpunar et al., 2008; Bäuml and Kliegl, 2013).

The present study addressed a third benefit of retrieval practice, which is referred to as the interference reduction effect in the following. It refers to the finding that retrieval practice of previously studied target information can insulate this information against retroactive interference from subsequently studied non-target information (Halamish and Bjork, 2011; Potts and Shanks, 2012; Bäuml et al., 2014). Retroactive interference describes the prominent finding that retention of previously studied target items is typically impaired when additional non-target items have been encoded between study and test (Müller and Pilzecker, 1900). Halamish and Bjork (2011), however, showed that, in healthy individuals, retrieval practice compared to restudy of the initially studied target items can dramatically reduce the target items’ susceptibility to retroactive interference and thus improve target recall on a final recall test. Following the episodic account of retrieval-based learning (Karpicke et al., 2014), the interference reduction effect has been attributed to enhanced list segregation processes in prior work (Abel and Bäuml, 2014; see also Halamish and Bjork, 2011; Kliegl and Bäuml, 2016). The assumption is that retrieval practice produces distinct context cues, which enable better discrimination between target and non-target lists and thus reduce the target items’ susceptibility to retroactive interference from the non-target items during final recall testing.

While there is evidence from recent cognitive work that the interference reduction effect is robust in healthy individuals, to the best of our knowledge, there is no study to date that addressed the effect in a clinical subject sample. Because enhanced interference susceptibility is regarded a major factor for memory impairment in clinical populations, including patients with stroke and patients with TBI (e.g., Shum et al., 2000; Cowan et al., 2004; Dewar et al., 2010), it is a highly important research question whether the interference reduction effect is also present in memory-impaired patient groups. The present study took a first step toward addressing the issue and examined whether retrieval practice of previously studied target information insulates this information against retroactive interference from subsequently studied non-target information in persons with stroke. We predicted that, in persons with stroke, the interference reduction effect is reduced in comparison to healthy individuals, or is even eliminated. This prediction arises because contextual processing has been shown to be potentially impaired after stroke (e.g., Kessels et al., 2002; Swick et al., 2006). Such deficits in contextual processing may impair discrimination between target and non-target lists and thus reduce or even eliminate the interference reduction effect in patients with stroke.

Both persons with stroke, 4.6 months post injury on average, and healthy controls participated in the present study. In each of two experimental sessions, participants learned a first list of items (pictures plus names of common objects) to be remembered for a final free recall test. Next, participants either were tested in a word-stem cued recall test on their memory for this list (retrieval practice condition) or restudied the list’s items (restudy control condition). After that, participants either studied a second list of items (interference condition) or fulfilled an unrelated distractor task (no-interference condition). Participants’ memory for the list 1 items (and list 2 items in the interference condition) was assessed in a final recall test. On the basis of the results from prior work, four expectations arose. First, persons with stroke should show generally impaired memory for both lists’ items compared to healthy controls (e.g., Snaphaan and De Leeuw, 2007). Second, both groups of individuals should show a retroactive interference effect for the restudied list 1 items (e.g., Dewar et al., 2007). Third, healthy controls should show a smaller retroactive interference effect for retrieval-practiced than for restudied list 1 items, reflecting the interference reduction effect in healthy individuals (e.g., Halamish and Bjork, 2011). Fourth and most important, on the basis of the view that the interference reduction effect reflects enhanced contextual processing (Halamish and Bjork, 2011; Abel and Bäuml, 2014), the effect in persons with stroke should be reduced, or even be eliminated, because contextual processing may be impaired after stroke (Kessels et al., 2002; Swick et al., 2006).

## Materials and Methods

### Participants

Twenty-two patients with stroke (mean age: 54.9, *SD* = 8.8, range: 41–67 years; 15 males), 4.6 month post injury on average (*SD* = 4.5, range: 1–18 months), and 22 age-matched healthy controls (mean age: 56.6, *SD* = 7.2, range: 39–65 years; 11 males) participated in the study. Two more patients were tested but were eliminated due to withdrawal of consent (1) and admitted substance abuse (1). Patient and control groups did not differ significantly in age, *t*(42) = 0.69, *p* = 0.493, or gender, *χ^2^*(1) = 1.50, *p* = 0.179. There was also no significant difference in the two groups’ educational attainment levels (academic studies: 6 patients vs. 9 controls; university-entrance diploma: 3 patients vs. 1 controls; general certificate of secondary education: 7 patients vs. 10 controls; certificate of secondary education: 6 patients vs. 2 controls), *χ^2^*(3) = 4.13, *p* = 0.248.

Patients were recruited at the Clinic for Neurological Rehabilitation at the Bezirksklinikum Regensburg, Regensburg, Germany. Patients with neurological (e.g., epilepsy, multiple sclerosis) or psychiatric conditions (e.g., depression, psychosis) other than stroke were excluded from the study. Further exclusion criteria for participation were severe or global aphasia, severe dysarthria, severe neglect, history of alcohol abuse, and history of psychoactive substance abuse. The sample of healthy controls consisted of spouses or life partners of the patients, employees of the institution, and persons recruited from the community. All participants spoke German as native language and reported normal or corrected-to-normal vision. No data on participants’ income or socioeconomic status were collected. The study was carried out in accordance with the recommendations of the local ethical review committee at Regensburg University Medical Center with written informed consent from all subjects. All subjects gave written informed consent in accordance with the Declaration of Helsinki. The protocol was approved by the local ethical review committee at Regensburg University Medical Center.

Neurological deficits in stroke patients were assessed in a pre-experimental session approximately one week before the first experimental session on the memory task. Degree of neurological deficits was quantified according to the 11-item National Institutes of Health Stroke Scale (NIHSS), which is designed to be a relatively simple and reliable diagnostic tool that can be administered by both neurologists and trained non-neurologists (Goldstein and Samsa, 1997). Stroke patients’ mean score of the NIHSS was 5.2 (*SD* = 3.2, range: 1–13, median: 5), indicating minor (scores 1–4: 12 patients) to moderate severity (scores 5–13: 10 patients) of stroke-related deficits. Concerning stroke classification, stroke was classified as ischemic in 13 patients and hemorrhagic in 7 patients; two patients had both ischemic and hemorrhagic strokes. Concerning stroke localization in the brain, the right hemisphere was affected in 10 patients, the left hemisphere was affected in 5 patients, and both sides of the brain were affected in 7 patients. In one patient in the no-interference group, the right hippocampus was affected. In no other patient, the medial temporal lobe was affected. Note that, because of small sample size and low statistical power, the present data were not analyzed as a function of stroke classification or stroke localization.

### Neuropsychological Assessments

In the pre-experimental session, two neuropsychological tests were conducted to assess verbal memory and verbal intelligence in both stroke patients and healthy controls. Assessing verbal memory, the Verbal Learning and Memory Test was used (VLMT; Helmstaedter et al., 2001), which is the German version of the Rey Auditory Verbal Learning Test (Schmidt, 1996). The target list consisted of 15 semantically unrelated words and was orally presented five times in a row with item presentation rate of 2 s, with each list presentation closely followed by an immediate free recall test (Tests 1-5). After Test 5, a second non-target list of 15 new words was presented and tested. Next, the target list was tested again both immediately after study of the non-target list (Test 6) and after a delay of 10 min (Test 7). Finally, a yes/no recognition test was conducted, which contained the 15 words from the target list, the 15 words from the non-target list, and 30 new words, and participants’ task was to identify the items from the target list (Test 8). The results of the VLMT are shown in **Table 1**. Compared to healthy controls, stroke patients showed smaller learning and memory effects, as indicated by the sum of absolute recall rates in Tests 1–5, but a larger interference effect, as indicated by the difference in recall between Tests 5 and 6. The patients also performed more poorly than the controls in the final recognition test, as measured by sensitivity (hits minus false alarms) in Test 8. Together, the results suggest impaired verbal memory and learning and enhanced interference susceptibility in stroke patients compared to healthy controls.

**Table 1:** Neuropsychological tests: comparison between groups, means and SDs.

Variable	Patients	Controls	Statistics
**VLMT:**			
Immediate Recall (Test 1)	5.82 (*SD* = 2.28)	7.05 (*SD* = 1.81)	*t*(42) = 1.98^†^
Total Recall (Σ Tests 1–5)	46.64 (*SD* = 11.51)	56.3 (*SD* = 8.44)	*t*(42) = 3.23^∗∗^
Interference (Test 5–Test 6)	2.14 (*SD* = 1.55)	1.09 (*SD* = 0.97)	*t*(42) = 2.68^∗^
Delay (Test 6–Test 7)	0.05 (*SD* = 1.46)	0.41 (*SD* = 0.80)	*t*(42) = 1.02
Recognition (Test 8: Hits–FA)	8.86 (*SD* = 5.41)	12.82 (*SD* = 2.52)	*t*(42) = 3.11^∗∗^
**WST:**			
Correct Responses	30.82 (*SD* = 4.78)	33.64 (*SD* = 3.75)	*t*(42) = 2.18^∗^
**WMS-R:**			
Forward Counting Score	10.32 (*SD* = 1.73)	9.45 *(SD* = 1.74)	*t*(42) = 1.65
Backward Counting Score	6.68 (*SD* = 1.76)	6.95 *(SD* = 1.94)	*t*(42) = 0.49

*VLMT, Verbal Learning and Memory Test; WST, Wortschatztest, German vocabulary test; WMS-R, Wechsler Memory Scale Revised; reliably different between groups: ^†^p < 0.10, ^∗^p < 0.05, ^∗∗^p < 0.01.*

Assessing verbal intelligence, the German vocabulary test (Wortschatztest, WST; Schmidt and Metzler, 1992) was administered in the delay period between Test 6 and Test 7 of the VLMT. The WST comprised 42 word sequences, each containing one real word (the target word) and five meaningless words (the non-target words). Participants’ task was to indicate the target word in each word sequence. In the literature, vocabulary tests like the WST have been suggested to provide an estimate for patients’ premorbid intelligence level. In fact, however, vocabulary tests have been shown to potentially underestimate patients’ premorbid intelligence level (Binkau et al., 2014). In the present study, the controls outperformed the patients in the WST (see **Table 1**), indicating relative impairment of verbal intelligence in stroke patients compared to healthy controls at study time, without necessarily indicating relative impairment of patients’ premorbid intelligence level.

Two more tests were conducted at the end of the first experimental session on the memory task. First, both the patients and healthy controls did forward and backward counting tasks, which were taken from the revised Wechsler memory scale (Wechsler, 2008), assessing participants’ short-term/working memory performance. The results of these tasks revealed no differences between patient and control groups, thus indicating comparable short-term/working memory functions in stroke patients and healthy controls (see **Table 1**). Second, only the patients were screened for dementia by using the Mini-Mental State Examination (MMSE; Folstein et al., 1975). Patients scored at least 25 out of 30 possible points on the MMSE (mean: 27.9, *SD* = 1.46, median: 28). According to conventional interpretation of MMSE scores, none of the patients thus showed evidence of a dementing illness (Lezak et al., 2004).

### Design of the Memory Task

The experiment used a 2 × 2 × 2 design with the factors of PRACTICE (restudy vs. retrieval practice), INTERFERENCE (no interference vs. interference), and GROUP (patients vs. controls). PRACTICE was manipulated within subjects. In one experimental session on the memory task, list 1 items were restudied after initial study (restudy condition), whereas in the other experimental session, list 1 items were retrieval practiced after initial study (retrieval practice condition); the assignment of conditions to sessions was counterbalanced across participants in both the patient and control groups. INTERFERENCE was manipulated between participants. After the restudy/retrieval-practice phase, half the participants studied a second list of items (list 2; interference condition), whereas the other half fulfilled an unrelated distractor task (no-interference condition). Allocation of participants to experimental groups was made randomly. No significant differences in any demographic (or neuropsychological) variables between experimental groups arose, both in the patient and the control groups, all *p*s* >* 0.05.

### Material of the Memory Task

Six different item sets of eight items each were prepared, with the items consisting of object names and pictures taken from the Snodgrass and Vanderwart (1980) item pool. The items of each set were chosen in such a way that each object’s name had a unique first letter within a set. Items’ first letters were not unique between sets; on average, 4.29 of the items of a set shared the same first letters with another set (*SD* = 0.83, range: 3–6, median: 4). The assignment of item sets to list 1 and list 2 was counterbalanced in the practice and interference conditions, and matched between the patient and control groups.

### Procedure of the Memory Task

**FIGURE 1:**
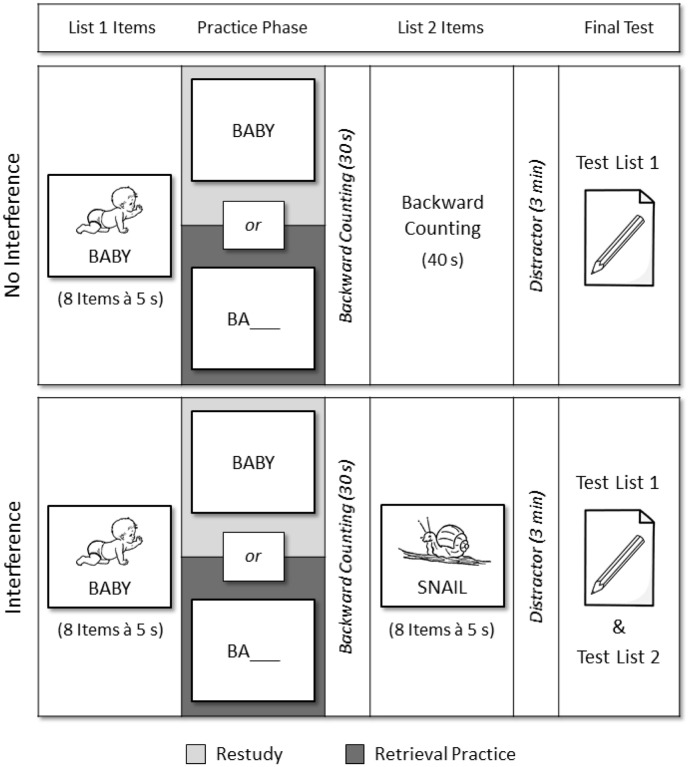
Procedure of the memory task. Stroke patients and healthy controls studied a first list of items (list 1) and underwent a restudy/retrieval-practice phase in which the list 1 items were either restudied or retrieval practiced. In the interference condition, participants studied a second list of items (list 2); in the no-interference condition, no list 2 learning took place. Participants’ memory for the two lists’ items was assessed in a final recall test.

Participants took part in two experimental sessions on the memory task, with the two sessions spaced approximately one week apart. In each session, participants studied a first list (list 1) of eight items, which they were asked to remember for a final recall test at the end of the session. Different list 1 (and list 2) items were used in the two sessions. The items were presented on 2.9 × 4.1 inch index cards in random order and with a presentation rate of 5 s. Black-on-white line drawings of the objects were depicted in the upper two thirds and the objects’ names were depicted in the lower third of the index cards (see **Figure 1**). The purpose of the drawings was to facilitate learning in the patient group. After initial study, the objects’ names were either restudied or retrieval practiced. In the restudy condition, index cards showing the objects’ names in the middle of the cards were presented in new random order with a presentation rate of 5 s. In the retrieval-practice condition, index cards showing two-letter word stems of the objects’ names in the middle of the cards were presented in new random order and participants were given 5 s to recall each item. All responses were given orally by the participants and were written down by the experimenter. After the restudy/retrieval-practice phase, participants counted backward in steps of ones from a three-digit number for 30 s.

Next, in the interference condition, participants studied a second list (list 2) of eight items, which they were asked to also remember for the final recall test at the end of the session. Participants were explicitly told that their memory for both list 1 and list 2 would be tested. List 2 items were presented on 2.9 × 4.1 inch index cards in random order and with a presentation rate of 5 s. Again, black-on-white line drawings of new objects were depicted in the upper two thirds and the objects’ names were depicted in the lower third of the index cards. In the no-interference condition, no list 2 was presented and backward counting was prolonged for 40 s instead. Following a 3 min distractor task, in which participants rearranged arrays of four single-digit numbers in an ascending order, the final recall test was conducted, in which participants were given unlimited time to recall in any order they wished the items from list 1; recall duration was not recorded by the experimenter. In the no-interference condition, participants were instructed to recall the items of the previously studied list; in the interference condition, they were asked to recall only the items of list 1. All responses were given orally by the participants and were written down by the experimenter. In the interference condition, list 1 recall was followed by a second free recall test in which participants were given unlimited time to recall the items from list 2; they were instructed to recall only the items of the second list.

Regarding list 1 recall, both correct recall and number of intrusions, i.e., number of list two items that were falsely recalled by participants in the final list 1 recall test, were examined. As it turned out, however, intrusions were produced very infrequently by participants (≤0.25 items per participant and condition), and therefore were not analyzed further. Regarding list 2 recall, correct recall was examined in the interference condition.

## Results

### Immediate List 1 Recall

Naturally, immediate list 1 recall in the cued recall test was assessed in the retrieval practice condition only. Immediate list 1 recall was generally high. It was numerically higher in healthy controls than in patients with stroke (96.0% vs. 89.8%), although the difference between the two groups did not reach significance, *t*(42) = 1.90, *p* = 0.065.

### Final List 1 Recall

**FIGURE 2:**
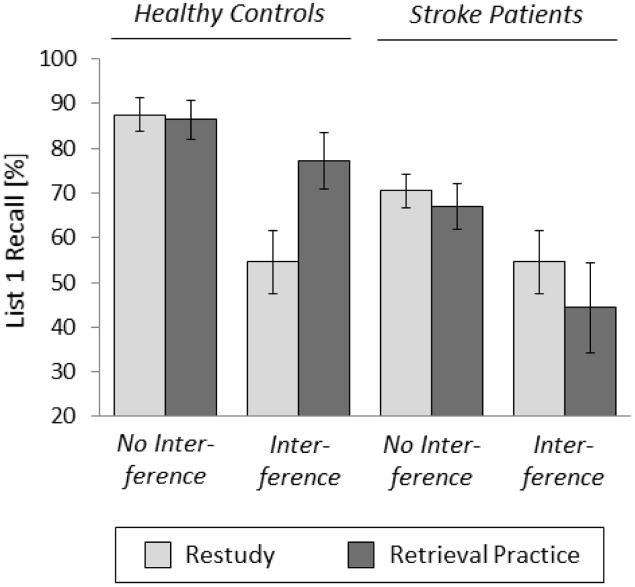
Mean recall rates of list 1 items as a function of PRACTICE (restudy vs. retrieval practice), INTERFERENCE (no interference vs. interference), and GROUP (controls vs. patients); error bars represent the standard error of the mean.

Final list 1 recall in the free recall test is shown in **Figure 2**. A three-way analysis of variance (ANOVA) with the factors of PRACTICE (restudy vs. retrieval practice), INTERFERENCE (no interference vs. interference), and GROUP (controls vs. patients) revealed significant main effects of INTERFERENCE, *F*(1,40) = 12.43, *p* = 0.001, partial η^2^ = 0.24, and GROUP, *F*(1,40) = 9.75, *p* = 0.003, partial *η*^2^ = 0.20. In addition, the three-way interaction between all three factors was reliable, *F*(1,40) = 4.77, *p* = 0.035, partial *η*^2^ = 0.11, which qualified the only remaining significant interaction between the factors of PRACTICE and GROUP, *F*(1,40) = 6.35, *p* = 0.016, partial *η*^2^ = 0.14. Indeed, other main effects and interactions were not significant, *F*(1,40)s < 1.40.

Based on the significant three-way interaction, separate two-way ANOVAs with the factors of PRACTICE (restudy vs. retrieval practice) and INTERFERENCE (no interference vs. interference) were calculated for the control and patient groups. For the control group, the analysis revealed a significant main effect of PRACTICE, *F*(1,20) = 4.53, *p* = 0.046, partial *η*^2^ = 0.18, a significant main effect of INTERFERENCE, *F*(1,20) = 7.88, *p* = 0.011, partial *η*^2^ = 0.28, and a reliable interaction between the two factors, *F*(1,20) = 5.59, *p* = 0.028, partial *η*^2^ = 0.22. Pair-wise comparisons showed that final list 1 recall was higher in the retrieval practice condition than in the restudy condition when retroactive interference from the learning of list 2 items was present (77.27% vs. 55.68%), *t*(10) = 2.79, *p* = 0.019, *d* = 0.84, whereas there was no such difference when retroactive interference was absent (87.5% vs. 86.4%), *t*(10) < 1. Indeed, the presence of retroactive interference reduced final recall of the restudied list 1 items, *t*(20) = 3.27, *p* = 0.004, *d* = 1.39, but had no significant effect on final recall of the retrieval-practiced list 1 items, *t*(20) = 1.20, *p* = 0.245, *d* = 0.51. The results indicate that retrieval practice insulated the list 1 items against retroactive interference from list 2 in healthy controls.

In contrast, for the patient group, the two-way ANOVA showed a significant main effect of INTERFERENCE, *F*(1,20) = 5.03, *p* = 0.036, partial *η*^2^ = 0.20, but neither a significant main effect of PRACTICE, *F*(1,20) = 2.05, *p* = 0.168, partial *η*^2^ = 0.09, nor a reliable interaction between the two factors, *F*(1,20) < 1. Indeed, final list 1 recall was higher when retroactive interference from list 2 was absent than when it was present, both in the restudy condition (70.5% vs. 54.5%), and the retrieval-practice condition (67.0% vs. 44.3%). These results indicate that retrieval practice did not insulate the list 1 items against retroactive interference from list 2 in patients with stroke.

### Final List 2 Recall

Naturally, final list 2 recall in the free recall test was assessed in the interference condition only. A two-way ANOVA with the factors of PRACTICE (restudy vs. retrieval practice) and GROUP (controls vs. patients) revealed a significant main effect of GROUP, *F*(1,20) = 4.98, *p* = 0.037, partial *η*^2^ = 0.20, but neither a significant main effect of PRACTICE nor a reliable interaction between the two factors, both *F*(1,20)s < 1. Indeed, the control group recalled more list 2 items than the patient group, both in the restudy condition (56.8% vs. 31.8%), and the retrieval-practice condition (62.5% vs. 36.4%).

## Discussion

This study examined the effects of retrieval practice and retroactive interference on retention of previously studied information, in both persons with stroke and healthy controls. Three major results emerged. First, persons with stroke compared to healthy controls showed generally impaired memory for both list 1 and list 2 items. Second, both patients and healthy controls showed a retroactive interference effect for the restudied list 1 items. Third and most important, healthy controls showed a relatively smaller retroactive interference effect for the retrieval-practiced than the restudied list 1 items, whereas the stroke patients showed the same detrimental interference effect for the retrieval-practiced list 1 items as they did for the restudied items. This finding indicates that retrieval practice failed to insulate list 1 items against retroactive interference from the learning of list 2 items in persons with stroke.

The results are consistent with the view that persons with stroke show impaired contextual processing (e.g., Kessels et al., 2002; Swick et al., 2006) and this impairment underlies the absence of an interference reduction effect in stroke patients in the present study. Indeed, on the basis of the view that enhanced contextual processing of the practiced material may underlie the retrieval-based interference effect (Halamish and Bjork, 2011; Abel and Bäuml, 2014), the impairment in contextual processing in patients with stroke should reduce or even eliminate the effect. The present pattern of retrieval-based interference reduction in healthy persons but no such reduction in stroke patients is in line with this reasoning.^1^

In the present study, no backward testing effect was observed. Indeed, list 1 recall in the no-interference condition did not reliably differ between retrieval-practiced and restudied items, both in stroke patients and healthy controls. The non-finding of a reliable testing effect when using a relatively short retention interval between practice and final test is well in line with the literature. In fact, while the testing effect has been shown to be a robust phenomenon when the final test is administered after relatively long retention intervals of days or weeks, the effect is typically absent or even reversed when the final test is administered after relatively short retention intervals of several minutes (e.g., Roediger and Karpicke, 2006; Toppino and Cohen, 2009). Using a relatively short retention interval of 3 min only, the present study thus is silent on whether a reliable testing effect can be present in patients with stroke.

Following the idea that the backward testing effect and the retrieval-based interference reduction effect are mediated by partially overlapping mechanisms, i.e., enhanced contextual processing of the practiced material, and on the basis of the present results, one may expect the backward testing effect to be reduced in patients with stroke as well. Such expectation may arise from the finding that the testing effect is typically present after longer, but not shorter delay (e.g., Roediger and Karpicke, 2006) and the view that longer retention intervals may increase extra-experimental interference (e.g., Baddeley et al., 2014). If so, both the interference reduction effect and the backward testing effect may reflect retrieval-based interference reduction and both be reduced, if not eliminated, in patients with stroke.

In contrast, on the basis of recent clinical work showing reliable backward testing effects in memory-impaired patient groups, one may expect the backward testing effect to be also present in patients with stroke. Reliable backward testing effects have been demonstrated in patients with Alzheimer’s disease, MS and severe TBI (Sumowski et al., 2010a,b; Small, 2012; Pastötter et al., 2013), and, with regard to semantic memory impairment, have also been shown in patients with stroke and chronic aphasia (Middleton et al., 2015; Friedman et al., 2017). On the basis of these finding and the present results, a dissociation between the backward testing effect and the interference reduction effect in patients with stroke may be expected, which would point to partially different mechanisms mediating the two effects. Future examination of the backward testing effect in patients with stroke may thus provide new insights into the mechanisms mediating different beneficial effects of retrieval practice.

Regarding list 2, the present results showed no reliable forward effect of testing. That is, list 2 recall in the interference condition was unaffected by whether list 1 items had been restudied or retrieval practiced, both in stroke patients and healthy controls. Arguably, the finding may be due to insufficient statistical power, as the present results showed at least a tendency toward a forward effect in the two subject groups. In addition, the effect may have been underestimated in the present study because, in contrast to the prior work on the forward effect, here list 1 recall preceded list 2 recall at final test (for related results, see Pastötter et al., 2012). Indeed, because, like the interference reduction effect, the forward effect of testing has been attributed to retrieval-based list segregation processes (Szpunar et al., 2008; Bäuml and Kliegl, 2013), on the basis of the present finding of no interference reduction effect in stroke patients after retrieval practice, one may expect the forward effect of testing to be also absent after stroke.

There are three potential limitations of the present study. First, sample size in the present study was small. Therefore, the study did not examine the impact of stroke classification or localization on the (absence of the) interference reduction effect. Future work that uses larger sample size and controls for factors of stroke classification and localization should therefore examine whether the (absence of the) effect depends on some specific neurological insult. Second, in the present study, patients’ memory was tested 4.6 months post injury on average. Because the memory impairment of stroke patients typically declines with increasing time after the stroke and is largely reduced one year after the stroke (Snaphaan and De Leeuw, 2007), it is a high priority for future work to examine to what extent patients’ failure to demonstrate the interference reduction effect is also reduced with increasing time after stroke. Arguably, one year after stroke, one may expect retrieval practice to insulate also stroke patients’ memory against interference. Third, the present study did not involve investigation of the neural effects of interference and retrieval practice in patients with stroke. Therefore, the study does not contribute to the understanding of the neural underpinnings of memory impairment and recovery after stroke. Future work is required to address this important issue.

In sum, the present results demonstrate that retrieval practice can insulate the tested information against retroactive interference from subsequently studied information in healthy persons, but fails to do so in patients with stroke. The finding has important implications for the understanding of long-term memory impairment after stroke. In the literature, enhanced interference susceptibility is considered a major factor for long-term memory impairment in patient groups, including patients with stroke. The present results thus suggest that stroke patients’ enhanced interference susceptibility may at least partly be due to the failure of retrieval practice to insulate episodic memories against retroactive interference. Future work is required to address the reliability and generalizability of the present results, employing more complex materials in more applied settings.

## Author Contributions

BP and K-HB developed the study concept. BP, K-HB, and HE contributed to the study design. HE collected the data. BP and HE performed the data analysis. BP drafted the manuscript, and K-HB, HE, and IA provided critical revisions. All authors approved the final version of the manuscript for submission.

## Conflict of Interest Statement

The authors declare that the research was conducted in the absence of any commercial or financial relationships that could be construed as a potential conflict of interest.

## Footnotes

1 Notably, the present results are also in line with the distribution-based bifurcation model (Halamish and Bjork, 2011; Kornell et al., 2011), which is a descriptive memory model of testing effects. The bifurcation model assumes that initially studied items are normally distributed on some memory-strength dimension. In particular, it assumes that the strength distribution for retrieval-practiced items is bifurcated between successfully retrieved and non-retrieved items, and the strength distributions are shifted differentially for retrieved and restudied items. Doing so, the model predicts that the presence of retroactive interference at test increases final test criterion and thus enhances the benefits of retrieval practice relative to restudy trials (for details, see Halamish and Bjork, 2011). The present results are consistent with the model, if we assume that strength distributions are shifted differentially for retrieved and restudied items in healthy controls, but are shifted to a similar degree in patients with stroke (for instance, because contextual processing during retrieval practice is impaired in persons with stroke).
